# Women in post-trafficking services in moldova: diagnostic interviews over two time periods to assess returning women's mental health

**DOI:** 10.1186/1471-2458-11-232

**Published:** 2011-04-14

**Authors:** Nicolae V Ostrovschi, Martin J Prince, Cathy Zimmerman, Mihai A Hotineanu, Lilia T Gorceag, Viorel I Gorceag, Clare Flach, Melanie A Abas

**Affiliations:** 1 N.Testemitanu Medical and Pharmaceutical University, Chisinau, Moldova; 2 Institute of Psychiatry, King's College London, London, UK; 3 International Organization for Migration, Chisinau, Republic of Moldova; 4 League of Mental Health, Moldova; 5 London School of Hygiene and Tropical Medicine, London, UK

## Abstract

**Background:**

Trafficking in women is a widespread human rights violation commonly associated with poor mental health. Yet, to date, no studies have used psychiatric diagnostic assessment to identify common forms of mental distress among survivors returning to their home country.

**Methods:**

A longitudinal study was conducted of women aged 18 and over who returned to Moldova between December 2007 and December 2008 registered by the International Organisation for Migration as a survivor of human trafficking. Psychiatric diagnoses in women at a mean of 6 months after return (range 2-12 months) were made by a trained Moldavian psychiatrist using the Structured Clinical Interview for DSM-IV, and compared with diagnoses recorded in the same women within 5 days of return. We described the socio-demographic characteristics of the women in the sample including both pre and post-trafficking information. We then described the distribution of mental health diagnoses recorded during the crisis intervention phase (1-5 days after return) and the re-integration phase (2-12 months after return). We compared diagnoses at the patient level between the two time points by tabulating the diagnoses and carrying out a kappa test of agreement and the Stuart-Maxwell test for marginal homogeneity (an extension of the McNemar test to kxk table).

**Results:**

120/176 (68%) eligible women participated. At 2-12 months after their return, 54% met criteria for at least one psychiatric diagnoses comprising post-traumatic stress disorder (PTSD) alone (16%); co-morbid PTSD (20%); other anxiety or mood disorder (18%). 85% of women who had been diagnosed in the crisis phase with co-morbid PTSD or with another anxiety or mood disorder sustained a diagnosis of any psychiatric disorder when followed up during rehabilitation.

**Conclusions:**

Trafficked women returning to their country of origin are likely to suffer serious psychological distress that may endure well beyond the time they return. Women found to have co-morbid PTSD or other forms of anxiety and depression immediately post-return should be offered evidenced-based mental health treatment for at least the standard 12-month period of rehabilitation.

## Background

Human trafficking is a human rights violation and modern form of slavery that occurs in and between most countries around the world. Trafficking in persons involves the movement of individuals by means of threat, force, coercion, or deception, for the purposes of exploitation or abuse [1]. The most commonly recognized form of human trafficking has been the sale of women and girls for sexual exploitation, but women, men and children are trafficked for various forms of labour, such as farming, manufacturing and begging [2]. Several countries in Eastern Europe, including Moldova, are well-known for high numbers of women who are trafficked for forced sex work [3]. Trafficked persons are frequently subjected to high levels of violence and abuse and at risk for symptoms of post-traumatic stress disorder (PTSD), depression and anxiety [4].

For survivors of trafficking, there are a growing number of centers around the world that offer post-trafficking care. Support services often include activities to address the mental health needs of individuals. Yet, while there are international calls for better psychological support, [5] there remains limited clinical evidence on the mental health needs of trafficking survivors. In one of the few studies exploring the mental health symptoms of women in post-trafficking assistance centers in Europe, findings based on the Brief Symptom Inventory [6] and the Harvard Trauma Questionnaire [7] indicate that within 0-14 days of entering care, the majority of women reported high symptom levels of PTSD, depression and anxiety [8]. Importantly, no studies to date have employed clinician-administered diagnostic assessment either to describe primary or co-morbid conditions. Notably, research among other groups suggests that co-morbidity in PTSD is common and may adversely affect prognosis [9].

According to conceptualization of the five stages of the trafficking process (pre-departure; travel and transit; destination; detention, deportation and criminal evidence; and integration and re-integration), [10] women will have different needs at different stages. This study focused on the last of these phases, re-integration, and women returning home. For the purposes of care and assistance the 'reintegration' phase may be divided into the 'crisis intervention' period, when urgent needs are met and support is aimed at safety and stabilization, and the 'rehabilitation' period, when support is aimed at longer-term recovery, rehabilitation and participation. This study aimed to: 1) describe women's mental health status, specifically psychiatric disorders, during the re-integration phase; and 2) explore changes in women's psychological symptoms over time, comparing diagnoses two to twelve months after their return, with earlier diagnoses made within five days of their return to Moldova.

## Methods

### Setting

The study took place in the Republic of Moldova in collaboration with the International Organization for Migration (IOM) in the Center for Assistance and Protection for Victims and Potential Victims of Trafficking in Human Beings. At the time of the study, IOM, as part of their Assistance, Prevention and Protection Programme, provided travel and reintegration assistance to individuals who chose to return or who were unable to remain in the country of destination http://www.iom.md/index.php/en/programs/counter-trafficking/assistance-a-protection-programme. Trafficked women in this study may have initially come into contact with IOM in destination settings or may have been referred to IOM in Moldova by a range of sources in destination countries, including police, immigration services, lawyers, health services and governmental and non-governmental anti-trafficking and support organizations.

### Profile of Re-integration Support System for Survivors of Trafficking

As part of their Assistance and Protection Programme (APP), IOM meets returning women who have been referred into their care at their port of entry. Between 2000 and 2008, IOM assisted 2340 women returned from a trafficking experience outside Moldova. IOM's support package for trafficked persons in Moldova is led by specially trained social workers, supported by an experienced psychologist (LG). Assistance generally consists of crisis intervention care, including a medical, psychological, legal and social needs assessment, and residential care of up to 1 month, which can be extended. This is followed by a 12-month community-based rehabilitation program, which often includes social assistance and vocational training. Approximately 80% of returning women accept the acute crisis intervention and/or the rehabilitation program. Mental health interventions include counseling, cognitive-behavioural therapy, antidepressant drug treatment, alcohol detoxification services and treatment for substance abuse and dependence. During rehabilitation, on-going antidepressant treatment relies on women being willing to attend a psychiatric hospital out-patient clinic.

### Sampling

We sampled consecutive women who registered with IOM between December 2007 and October 2008 and had participated in a crisis intervention psychiatric assessment within 5 days of registering. We approached them between February 2008 and December 2008, at least two months after their registration.

### Recruitment

Women survivors of trafficking were approached by an IOM social worker two to twelve months after return, either face to face or by phone, generally during their monthly follow-up appointment in the rehabilitation programme. Women were informed of the study aims, subject matter and the voluntary nature of participation was emphasised. Women who gave permission to be approached by the research team and who consented to be interviewed were interviewed at the Rehabilitation Centre or at another place of their choosing. We followed the World Health Organization Ethical and Safety Recommendations for Interviewing Trafficked Women [11]. The process complied with the IOM Data Protection Principles [12]. Ethical approval was obtained from Kings College Research Ethics Committee (CREC/07/08-56) and from N.Testemitanu State Medical and Pharmaceutical University Institutional Review Board.

### Inclusion criteria

Women were included if they were aged 18 or over, originally resident in Moldova, had returned to Moldova in the past 2-12 months following a trafficking experience outside of Moldova, registered with IOM in Moldova as a survivor of trafficking and participated in the crisis-intervention assessment (described above) 2-12 months prior. IOM defined all survivors of trafficking according to UN Protocol to Prevent, Suppress and Punish Trafficking in Persons, especially Women and Children [1].

### Exclusion criteria

Women were not invited to participate or were excluded if the social worker or research psychiatrist considered them to be too distressed or unwell to take part.

### First data collection; Crisis intervention period and the initial psychiatric assessment

Clinical assessments of women's initial psychiatric conditions were made available to the researchers, for women who consented. These initial psychiatric assessments were conducted within two days of women's arrival at the IOM centre (and within 5 days of registering with IOM as a returning trafficked person) by a senior consultant psychiatrist (MH), based on the International Classification of Diseases (ICD-10) [13]. A semi-structured assessment was carried out using a locally developed checklist, which included items such as, history of feelings or symptoms of emotional distress or mental health or medical problems, current and past psychiatric and medical treatment, history of illicit drug use, nicotine and alcohol use, current medication, family background and family history of illness, personal history (which included information on early development, school/work, relationships, current life circumstances and interests), premorbid personality, a mental state examination.

Socio-demographic variables were available from recorded data, which included marital status before trafficking, employment status prior to trafficking, country trafficked to; age on return from trafficking. IOM provided restricted access to limited data in anonymised aggregated form on women who did not take part in order to enable broad comparisons to be made between participants and non-participants. The process fulfilled to the IOM Data Protection Principles [14].

### Second data collection - rehabilitation period psychiatric assessment

Participants were interviewed by a Moldovan psychiatrist using the operational criteria of the DSM-IV two to twelve months after having returned to Moldova. This timeframe was selected based on staff's assessment that by this time, women's urgent needs had been met, and they were generally beginning to adjust and resettle and 12 months was the normal maximum period of support provided by the Rehabilitation Centre.

We used the Romanian version of the Non-Patient version of the Structured Clinical Interview for DSM-IV Axis I Disorders [15]. The diagnoses for the rehabilitation period interviews were made by a Moldavian psychiatrist (N.O.), familiar with the operational criteria of the DSM-IV and trained in the SCID (Video course). We assessed current (last month) mental illnesses according to the Diagnostic and Statistical Manual of Mental Disorders, fourth edition [16]. We defined having a psychiatric illness as fulfilling criteria for at least one DSM-IV Axis I psychiatric disorder. We considered translating the Schedules for Clinical Assessment in Neuropsychiatry [17] in order to be able to make ICD diagnoses, but determined that this would have been prohibitively expensive and time consuming. The SCID translated version was pre-tested by two Moldavian psychiatrists trained in standardised interviews (MH, NO) and found to be understandable to the women and of good face validity.

Alcohol use was assessed with the Alcohol Use Disorders Identification Test (AUDIT) [18], a 10-item screening instrument measuring hazardous and harmful alcohol consumption which covers consumption, drinking behaviour and alcohol related problems. Total scores of 8 or more were considered as indicators of hazardous and harmful alcohol use, as well as possible alcohol dependence [19]. Substance abuse/dependence was measured using five questions based on the Diagnostic Interview Schedule about the frequency of drug use, stated dependence, inability to cut down, need for larger amounts, and withdrawal symptoms. A list of drugs used in Moldova was provided, including cannabis, amphetamines, opiates, hallucinogens, ecstasy and solvents. Those who reported the use of any of the listed substance within the previous month were regarded as having a substance abuse. Those who answered positively to one of the questions about inability to cut down need for larger amounts, and withdrawal symptoms were classified as dependent. As the method of first and second data collection approaches differed slightly, the disorders are grouped at a higher level to be comparable - see Analysis.

### Sample size calculation

Data with a convenience sample of 200 trafficked women located across seven European countries and in touch with services suggests that approximately 50% of women recently returned from being trafficked may have a depressive or anxiety disorder [8]. To estimate this prevalence with 95% confidence intervals, +- 7.5%, at 90% power, we needed a sample of 120 women.

### Analysis

All analyses were carried out in Stata version 10 [20]. We described the socio-demographic characteristics of the women in the sample including both pre and post-trafficking information (See Table 1). We then described the distribution of mental health diagnoses recorded during the crisis intervention phase (1-5 days after return) and the re-integration phase (2-12 months after return. We compared diagnoses at the patient level between the two time points by tabulating the diagnoses and carrying out a kappa test of agreement and the Stuart-Maxwell test for marginal homogeneity (an extension of the McNemar test to kxk table). These will test for the level of agreement in diagnosis of the women at the two time points and for symmetry in the cross-tabulation of diagnoses to determine if there is any systematic change in diagnoses or if changes occur evenly. Since the diagnostic tools used in the two time periods were different, in order to compare between them we grouped specific conditions into four broad syndrome clusters; PTSD; anxiety disorders excluding PTSD (panic disorder, generalized anxiety disorder and somatisation disorder); mood disorders (depression and dysthymia); and substance use disorders (substance abuse, substance dependence, alcohol use and alcohol dependence). Those with a diagnosis of adjustment or acute stress disorder during the crisis intervention phase were not included in the statistical test for change in diagnosis because these time-limited diagnoses would be very unlikely to be diagnosed at the later time point and would therefore be inappropriate for comparison.

**Table 1:** Socio-demographic characteristics for women survivors of trafficking.

Age (years)	n (%)
18-20	21(17.5)

21-25	59 (49.2)

26-30	14 (11.7)

31-45	26 (21.7)

Marital status before trafficking	

Single	82 (68.3)

Married/Co-habiting	14 (11.6)

Separated/Divorced/widowed	24 (19.9)

Education	

Primary education or less	14 (11.6)

Lower secondary (compulsory 9 years)	75 (62.5)

Upper secondary or more	31 (25.7)

Employment prior to trafficking	

Unemployed	82 (68.2)

Unqualified work	25 (20.8)

Student/vocational training	7 (5.8)

Qualified work	6 (5)

Family contact post-trafficking	

One/both parents alive remain in contact	91 (75.6)

One/both parents alive none in contact	24 (19.8)

Both deceased	5 (4.1)

Current Residency	

Rural	81 (67.5)

Urban	39 (32.5)

Country trafficked to	

Turkey	47(39.7)

Russia	33 (27.5)

EU countries	14 (11.6)

Other (e.g. Kosovo, Albania)	26 (21.2)

Employment post-trafficking	

Unemployed	44 (36.6)

Unqualified work	37 (30.8)

Student/vocational training	25 (20.8)

Qualified work	14 (11.6)

Confidant prior to trafficking	

Yes	51 (42.5)

No	69 (57.5)

Confidant post-trafficking	

Yes	39 (32.5)

No	71 (67.5)

Time period in trafficking situation (months)	

1-3 months	11 (9.2)

4-6 months	28 (23.3)

7-12 months	48 (40.0)

13-24 months	30 (25.0)

More than 24 months	3 (2.5)

## Results and Discussion

**Figure 1:**
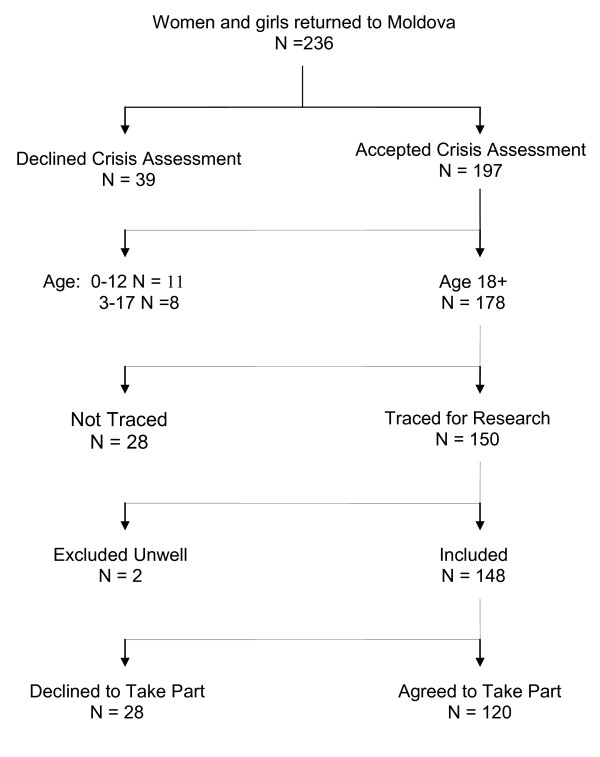
**Study Flow chart**.

During the study period, between December 2007 and December 2008, 178 women aged 18 and over were registered with IOM and participated in crisis assessment. See Figure 1. Social workers were subsequently able to trace 150 of these women, of whom two were excluded because of on-going severe physical illness. Of the 176 eligible, nine declined to be approached by the research team, 19 declined to give informed consent after being approached by the research team, and 28 we were unable to trace. Follow-up assessments were ultimately completed for 120 of the 176 women. More than a third (40%) of the interviews took place at the IOM Rehabilitation Center and the majority (60%) took place at various locations chosen by the women, e.g. their home, nearest hospital or the regional social work office. The mean time for the follow-up interview was 6 months after the crisis interview with 65% of women interviewed between 3 and 8 months.

Based on the limited demographic data available to compare participants and non-participants, very little difference was observed. For example, the mean age of non-participants is 24 years-old, versus 25 for participants, 10.0% of non-participants had primary education or less compared to 11.6% of participants; and 67.1% of non-participants were single before being trafficked versus 68.3% of participants.

### Characteristics of women returned after being trafficked

As shown in Table 1, most women were single before being trafficked and most had completed compulsory lower secondary education (nine years), although 12% had only attended primary school. The mean age for the sample at return was 25 years and nearly one fifth was younger than 21. Forty-seven percent of women had at least a child. Before being trafficked, 68% had been unemployed, however, this decreased to 37% post-trafficking, mostly due to opportunities for study or training organized through the IOM rehabilitation program. For 68%, the trafficking situation lasted for more than six months. Since return, a fifth were not in contact with either of their parents, and less than a third of women reported having someone in whom they thought they could confide.. Most were living rurally after return.

### Total prevalence of psychiatric illnesses 1-5 days after return and 2-12 months after return

Table 2 shows the total prevalence of all psychiatric illnesses, allowing for the possibility that the total number of diagnoses may be more than the number of women. In the crisis intervention period, the most common diagnoses were anxiety disorders (especially PTSD, diagnosed in 48% of women), mood disorders, adjustment and acute stress disorders and substance use disorders. Two to twelve months later during the rehabilitation period, the most common diagnoses were PTSD, mood disorder or harmful alcohol use.

**Table 2:** Total Prevalence of psychiatric diagnoses in 120 Moldavian women returned to Moldova after being trafficked

Disorder	Psychiatric illness at crisis intervention phase (1-5 days after return)	Psychiatric illness at re-integration phase (2-12 months after return)
	N of diagnoses/%	N of diagnoses/%

**Anxiety disorders**		

Posttraumatic stress disorder	58 (48.3)	43 (35.8)

Panic disorder	4 (3.3)	5 (4.1)

Generalized anxiety disorder	4 (3.3)	8 (6.6)

Somatisation disorder	3 (2.5)	0 (0)

Any anxiety disorder	69 (57.5)	56 (44.2)

**Mood disorders**		

Major depression	4 (3.3)	24 (16.7)

Dysthymia	4 (3.3)	5 (4.1)

Any mood disorder	8 (6.6)	29 (24.1)

**Adjustment disorder**	16 (13.3)	0 (0)

**Acute stress reaction**	12 (10)	0 (0)

**Substance use disorders**		

Alcohol harmful use^a, ^or abuse^b^	5^a ^(4.2)	10^b ^(8.3)

Alcohol dependence	8 (7)	5 (4.2)

Substance harmful use a/abuse b	4 (3.3)	5 (4.2

Substance dependence	7 (5.8)	4 (3.3)

Any alcohol or substance use disorder	24 (18.3)	24 (18.3)

**Psychosis**		

Paranoid Schizophrenia	2 (1.6)	0 (0)

**Total number of diagnoses**	131 (110)	99 (82.5)

^a ^according to ICD-10 classification - harmful use

^b ^according to DSM-IV classification - abuse

### Diagnostic profile for individual women 1-5 days after return and 2-12 months after return

Table 3 shows the principal single or co-morbid diagnosis for each woman. It shows that the level of co-morbidity is high in the sample at both time periods. During the rehabilitation period, 16% of women had 'pure' PTSD, while 20% had PTSD plus at least one secondary diagnosis. In more detail, (not shown in the table), 7% of women at rehabilitation had PTSD co-morbid with depression or dysthymia, 3% of women had PTSD co-morbid with another anxiety disorder, 5% had PTSD co-morbid with substance misuse (alcohol or drug misuse), and 6% had PTSD co-morbid with both substance misuse and a mood or other anxiety disorder.

**Table 3:** Diagnostic profile (principal single or co-morbid psychiatric diagnosis) for 120 Moldavian women returned to Moldova after being trafficked

	Psychiatric illness at crisis intervention phase (1-5 days after return) (ICD-10)	Psychiatric illness at re-integration phase (2-12 months after return) (DSM-IV)
	N of women, %	N of women, %
	N = 120	N = 120

No diagnosis	15 (13)	55 (46)

Adjustment or acute stress disorder	28 (23)	0 (0)

Posttraumatic stress disorder	42 (35)	18 (15)

PTSD co-morbid with mood, other anxiety, or substance use disorder.	16 (13)	25 (21)

Mood and/or anxiety disorder (not PTSD),	11 (9)	12 (10)

Mood or anxiety disorder co-morbid with substance use disorder or other (not PTSD), add to above	5 (4)	10 (8)

other	3 (3)	0 (0)

Total with a diagnosis	105 (88)	65 (54)

### Course and outcome of psychiatric illness

As shown in Table 4, of those women having co-morbid PTSD or another anxiety or mood disorder at the early crisis intervention phase, 85% had a psychiatric illness diagnosed later, during rehabilitation. However, for those with pure PTSD or with adjustment or acute stress disorder only 1-5 days after return, only 40% had a diagnosable psychiatric illness at rehabilitation. The kappa test for agreement and Stuart-Maxwell test for symmetry were run on 92 patients who did not have a diagnosis of adjustment or acute distress disorder at the crisis intervention phase. The reported diagnoses gave a kappa level of 0.27 and 41% agreement indicating low agreement between the time points. The test of homogeneity was significant (chi2 = 27.17, df = 5, p < 0.001) indicating that the changes in the diagnoses of the women are not equally distributed but systematically differ between the two time points, for example the large proportion that have moved from PTSD to no-diagnosis.

**Table 4:** Outcome of psychiatric illness for 120 Moldavian women returned to Moldova after being trafficked *

	Psychiatric illness at re-integration phase (2-12 months after return)*					(DSM-IV)	
Psychiatric illness at crisis intervention phase (1-5 days after return)* (ICD-10)	**No diagnosis**	**PTSD**	**PTSD+co-morbid**	**Mood or anxiety (not PTSD)**	**Mood or anxiety +co-morbid (not PTSD)**	**Other**	**Total**

No diagnosis	9 (10%)	1 (1%)	0 (0%)	4 (4%)	1 (1%)	0 (0%)	15(16%)

PTSD	25 (27%)	9 (10%)	8 (9%)	0 (0%)	0 (0%)	0 (0%)	42(46%)

PTSD+co-morbid	3 (3%)	2 (2%)	11 (12%)	0 (0%)	0 (0%)	0 (0%)	16(17%)

Mood or anxiety (not PTSD)	1 (1%)	1 (1%)	2 (2%)	5 (5%)	2 (2%)	0 (0%)	11(12%)

Mood or anxiety + co-morbid (not PTSD)	0 (0%)	0 (0%)	1 (1%)	0 (0%)	4 (4%)	0 (0%)	5 (5%)

Other	0 (0%)	2 (2%)	0 (0%)	1 (1%)	0 (0%)	0 (0%)	3 (3%)

Subtotal	38 (41%)	15 (16%)	22 (24%)	10 (11%)	7 (8%)	0 (0%)	92

Adjustment or acute stress disorder	17 (61%)	3 (11%)	3 (11%)	5 (18%)	0 (0%)	0 (0%)	28

Total	55 (46%)	18(15%)	25 (21%)	15 (13%)	7 (6%)	0 (0%)	120

* Percentages in table are cell percentages

## Discussion

This is the first study to evaluate the mental health of trafficked women using clinician-administered psychiatric diagnostic assessment. In a consecutive cohort of women returning to Moldova who were in contact with post-trafficking care services, we found that most (88%) women emerged from trafficking experiences with significant psychological distress, and that a portion (54%) reached levels of clinical diagnoses for common mental disorders at 2-12 months after return. We also found that women diagnosed with co-morbid PTSD or with another anxiety or mood disorder shortly after their return were likely to continue to have poor mental health later, with 85% of such women sustaining a diagnosis of any psychiatric disorder over the following 2-12 months.

The high rate of psychiatric illness identified at the re-integration period is most likely explained by the serious nature of trauma experienced by the women during the trafficking experience. Human trafficking, especially trafficking for sexual exploitation, is renowned for the extreme forms of abuse and intimidation experienced by trafficked persons, including sexual violence, physical violence, threats of harm to themselves and their family and severely restricted movements[21]. Many of the tactics used by traffickers may be compared to those used in situations of torture [22] and are often associated with high levels of PTSD, depression and anxiety [23-26]. Other factors explaining the high rate of chronic mental disorder may include childhood adversity, personality factors, and socio-economic position ([27]. In future papers we will present analyses that explore the impact of such factors in predicting lack of mental health recovery between baseline and follow-up.

We waited a minimum of 2 months before the follow-up interview and a maximum of 12 months. The mean time of follow-up interview was 6 months with 65% of women interviewed between 3 and 8 months. After one month, over 90% of women have left the crisis centre and are living in the community, supported by social assistance and vocational training. It is possible that women were facing a large number of stressors at this period, which could contribute to the high prevalence of mental distress. Women returning may face a myriad of difficulties after returning including family difficulties, poverty, stigma and social isolation, which pose serious challenges for their recovery. It is possible if women had been interviewed later, after 12-15 months, that we might have seen more adjustment and recovery.

We found a high rate of co-morbidity of mental disorders in re-integration phase, with the most common diagnosis being PTSD co-morbid with depression, another anxiety disorder, or with alcohol or substance use disorder. This is consistent with previous evidence suggesting that co-morbidity is common in trauma victims, especially PTSD with depression, [28,29] with one review concluding that in many cases, depression and PTSD are related but independent sequelae of trauma [9].

We also found that women with a diagnosis of co-morbid PTSD, compared to PTSD alone, at the early crisis intervention phase were especially likely to have a psychiatric illness 2-12 months later. Co-morbidity may partly reflect severity of the condition, which is known to be a predictor of worse outcomes [9]. Factors such as pre-departure abuse, [10] severity of trafficking abuse, length of time in the trafficking situation [21] or low levels of social support [30] may influence susceptibility to co-morbid diagnoses. It is worth recalling that less than a third of women reported feeling able to disclose to someone. Women's symptom patterns may also reflect, in part, the treatment approach, which included primarily anti-depressant drugs (which may have been inadequate), and women may have responded better if they received trauma-focussed cognitive-behavioural therapy [31] or other evidenced-based treatment, such as narrative exposure therapy [32] or eye movement desensitization and reprocessing, which are not currently available in Moldova.

### Limitations

Study participants were all registered with the International Organisation for Migration (IOM) and recruited via contact with their social worker. This may mean that women with a higher level of need are represented versus if we had attempted to include women not in contact with services, biasing the sample towards overestimating mental distress. However, women not included in the study may have been those who were too distressed to identify and access services or experienced other barriers to service access such as poor education. Given the highly sensitive nature of trafficking, we did not consider it appropriate to approach women who were not contactable through a known service [11]. It is worth noting, however, that very few differences were found between women who participated versus those who did not. We were able to recruit 68% of consecutive women returning through IOM's APP program, supporting our view that our sample is reasonably representative of women participating in IOM's rehabilitation program.

It is unclear to what extent findings from this study may be generalisable to the broader population of trafficked women. Participants had each been referred into care and therefore may differ from the larger population of trafficked women that do not access assistance. It is likely to be only the smallest portion of women who are trafficked who receive post-trafficking support, and there is, to date, no data to compare this sample with what might be considered a 'general population of trafficked persons. For this study, we also do not have information on the differences in mental health support that women received during the rehabilitation period, so we can not account for any interventions that may have influenced women's conditions over two time periods.

A further limitation may be the different instruments used at the two time periods evaluated. The ICD-10 [13] classification was used at when women entered in the crisis phase, in line with normal practice in the centre, and DSM-IV [16] was used at the re-integration phase. Although there are at least minor differences between two classifications in almost every category of disorder, these two widely used classifications are generally found to be functionally equivalent [33] and are largely comparable[34] with a high level of concordance reported for depression, dysthymia, substance dependence and generalised anxiety disorder [33]. For PTSD, concordance between ICD and DSM is reported to range from 35% to 75% [33,35]. One key difference is that ICD has a potentially lower threshold for experiencing a severely threatening event (such as being in a war zone whereas DSM requires the person to have experienced threat to their life of themselves or others to which they reacted with helplessness and/or horror. The second key difference is that ICD does not require impairment to be present whereas DSM does. The third difference is that DSM requires three symptoms of avoidance whereas ICD requires only one. Overall, the effect o the differences is that using the ICD classification is recognised to lead to an increase in prevalence estimates of PTSD, compared to if DSM criteria are used, with up to a doubling of prevalence of PTSD being reported from ICD versus DSM. Given this, our methodological differences are more likely to explain any apparent recovery by the second time period [33]. As our key finding is the extent of on-going psychiatric illness in the second time period for those with co-morbid PTSD, anxiety or depression at baseline, we can be confident that women with diagnoses at the rehabilitation phase had significant levels of mental distress and impairment, and that any difference between the two methods cannot account for women remaining or becoming more mentally unwell at follow-up. We used DSM at follow-up because a standardised interview for DSM was available in the local languages and also because DSM is well-accepted as portraying the current theoretical construct of PTSD [33,35].

### Implications for research

There is currently limited knowledge about effective treatment for PTSD co-morbid with depression, especially in victims of extreme trauma. Studies are needed to evaluate treatments that show promise, such as narrative exposure therapy [32,36] and trauma-focussed cognitive behaviour therapy with and without pharmacotherapy, [31] particularly in populations of trafficked persons.

## Conclusions

Post-trafficking support should include mental health assessment and care both in the crisis and the rehabilitation periods. Practitioners should be especially vigilant during the crisis period for women with diagnosable anxiety or mood disorder or co-morbid PTSD, who will be at risk of on-going serious disorders. Services should also be prepared to provide or refer women to detoxification or addiction services for substance misuse problems. As states establish or refine assistance measures for trafficking survivors, including transnational and national referral mechanisms, [37] policy-makers should ensure that adequate funds are dedicated to support healthcare programs that include long-term mental health care.

Caution must be used to avoid burdening trafficked women with additional stigmatizing labels by diagnosing them with a 'disorder' given that in many cases, their psychological reactions to such life-threatening violations are normal responses to extraordinarily abnormal events. Approaches to assessment and care should prioritize confidentiality, sensitivity and empowerment to offer women the greatest hope of recovery and a better future.

## Competing interests

The authors declare that they have no competing interests.

## Authors' contributions

NO and MA were responsible for designing the study and selecting study instruments with support from MP, MH and CZ. NO, LG, VG collected the data. MA and MH supervised the study with support from MP. NO, MA, and CF analyzed the data. NO wrote the first draft of the paper, MA wrote the second draft. CZ, VG, MH, LG and CF contributed to subsequent drafts and all authors contributed to the final draft and approved the final draft.

## Pre-publication history

The pre-publication history for this paper can be accessed here:

http://www.biomedcentral.com/1471-2458/11/232/prepub
